# The VPS4 component of the ESCRT machinery plays an essential role in HPV infectious entry and capsid disassembly

**DOI:** 10.1038/srep45159

**Published:** 2017-03-28

**Authors:** Justyna Broniarczyk, David Pim, Paola Massimi, Martina Bergant, Anna Goździcka-Józefiak, Colin Crump, Lawrence Banks

**Affiliations:** 1 Tumour Virology Laboratory, International Centre for Genetic Engineering and Biotechnology, Padriciano 99, Trieste, I-34149, Italy; 2 Department of Molecular Virology, Adam Mickiewicz University, Umultowska 89, Poznan, 61-614, Poland; 3 Centre for Biomedical Sciences and Engineering, University of Nova Gorica, Vipava, Slovenia; 4 Division of Virology, Department of Pathology, University of Cambridge, Tennis Court Road, Cambridge, CB2 1QP, UK

## Abstract

Human Papillomavirus (HPV) infection involves multiple steps, from cell attachment, through endocytic trafficking towards the trans-Golgi network, and, ultimately, the entry into the nucleus during mitosis. An essential viral protein in infectious entry is the minor capsid protein L2, which engages different components of the endocytic sorting machinery during this process. The ESCRT machinery is one such component that seems to play an important role in the early stages of infection. Here we have analysed the role of specific ESCRT components in HPV infection, and we find an essential role for VPS4. Loss of VPS4 blocks infection with multiple PV types, suggesting an evolutionarily conserved critical step in infectious entry. Intriguingly, both L1 and L2 can interact with VPS4, and appear to be in complex with VPS4 during the early stages of virus infection. By using cell lines stably expressing a dominant-negative mutant form of VPS4, we also show that loss of VPS4 ATPase activity results in a marked delay in capsid uncoating, resulting in a defect in the endocytic transport of incoming PsVs. These results demonstrate that the ESCRT machinery, and in particular VPS4, plays a critical role in the early stages of PV infection.

Human Papillomaviruses (HPVs) are the causative agents of a number of prevalent human cancers, with cervical cancer being the most important. Over 200 different HPV types have been described, the vast majority of which cause only benign lesions^1^,^2^. However, a small subset of 12 HPV types have been defined as cancer-causing in the cervix^3^. A critical feature of the HPV life cycle is its intimate link to the differentiation of the infected epithelium. These viruses are believed to gain entry into the basal keratinocytes through microtraumas in the skin. Then as these basal cells divide and begin terminal differentiation, there is coordinate expression of the different viral gene products, ultimately resulting in the production of new infectious viral particles^4^. Essential players in this whole process are the E6 and E7 oncoproteins, which create an environment favourable for viral DNA replication within the mid-epithelial layers, where under normal circumstances DNA replication would not be possible^5^. In rare cases this infection cycle is perturbed and changes occur, which can ultimately give rise to the development of a cancer^6^.

The HPV capsid consists of two viral proteins, the major component L1, and the minor component L2^7^. Whilst L1 plays an essential role in maintaining the structural integrity of the capsid, the L2 protein appears to play a major role in ensuring that the incoming viral genome is trafficked correctly to the nucleus, where viral gene expression can initiate^8^. Infection of the target keratinocyte by HPV appears to be a highly complex process. Initial attachment to the basement membrane is believed to occur through association with heparan sulphate proteoglycans^9^,^10^ and, following a conformational change to the viral capsid, which allows furin cleavage of the L2 N-terminus, the virus binds to an as yet unidentified receptor on the target cell^8^,^11^. The virus is then endocytosed by a rather complex and somewhat controversial process^12^ and, then following endocytic transport and acidification, there is progressive capsid uncoating. Finally the L1 and L2 proteins are separated through the action of cyclophilins^13^, resulting in L1 degradation in the lysosomal compartment^14^,^15^. The L2 protein is believed to be partially exposed to the cytosol relatively early during the infectious process^16^,^17^ and then, L2 recruits components of the endocytic sorting machinery, including the retromer, Sorting Nexin 17 (SNX17) and SNX27^18^,^19^,^20^,^21^ which together support the transport of the L2-DNA complex to the trans Golgi network^22^. During mitosis there is membrane dissolution and nuclear envelope breakdown, which allows the L2-DNA complex to enter the nucleus and initiate viral gene expression at PML oncogenic domains^23^,^24^,^25^,^26^.

Whilst many of the steps in virus entry are beginning to be characterized, there are still major questions about the roles of specific stages of endosomal maturation, and in particular that of the formation of multi-vesicular bodies (MVBs) and the associated components of the endosomal sorting complex required for transport (ESCRT) complex that are required for MVB formation^27^,^28^,^29^. Analysis of the location of incoming HPV capsids had suggested their presence within MVBs^12^, and studies had also shown a potential requirement for the ESCRT component TSG101 for efficient virus entry^30^. More recently, the ESCRT associated adaptor protein ALIX, was also shown to be required for intracellular trafficking of incoming HPVs and subsequent capsid disassembly^31^. The ESCRT machinery is highly complex, involving multiple steps and many different cellular proteins. The pathway is broadly defined as comprising four complexes, ESCRT0, I, II, III, with different cellular proteins being representative of each stage^32^. For example TSG101 is part of the ESCRTI complex and HRS defines ESCRT0. The VPS4 protein, which has two isoforms, VPS4A and VPS4B, belongs to the family of AAA-ATPAses, which like ALIX, are considered to be ESCRT accessory proteins recruited from the cytosol. Through its ATPase activity, VPS4 plays an important role in the recycling of the ESCRTIII components and the maintenance of MVBs^33^,^34^. In order to gain more insight into the potential role of the ESCRT machinery and MVB formation in the HPV entry pathway we investigated the potential requirements for HRS and VPS4 for HPV infection. Here we demonstrate a critical requirement for the VPS4 ATPase, without which there are major defects in HPV infectious entry. This appears to be mediated through a defect in capsid uncoating, resulting in a block to the normal trafficking of L1 and L2.

## Materials and Methods

### Cell lines and transfections

HaCaT, HEK293 and HEK293TT^35^ were maintained in DMEM supplemented with 10% fetal bovine serum, penicillin-streptomycin (100 U/ml) and glutamine (300 mg/ml). Ecdysone responsive HEK293 cells expressing inducible GFP tagged VPS4 wild type and VPS4EQ mutant were maintained in 400 ug/ml Zeocin and 800 ug/ml G418 and VPS4 expression induced by treating with 1 uM ponA (Ponasterone A, Invitrogen) for 24 h as described previously^36^. For transient siRNA experiments the cells were transfected using Lipofectamine RNAiMAX (Invitrogen) with siRNAs against TSG101, VPS4A, VPS4B, HRS (ON-TARGETplus SMARTpools, Dharmacon) or non-targeting siRNA (scrambled siRNA) as a control. For HEK293 cell transfections calcium phosphate precipitation was used37.

For HEK293 cell transfections calcium phosphate precipitation was used^37^.

### Plasmids

The plasmids expressing the HPV-16 L1 and HPV-16 L2 GST fusion proteins have been described previously^20^. GFP tagged VPS4A and DsRed tagged VPS4B have been described previously^38^ and were kindly provided by Wesley Sundquist. His-tagged VPS4B expression plasmids were provided by Wesley Sundquist and John McCullough. HRS-pEGFP-C plasmid was kindly provided by Clarisse Berlioz-Torrent and has also been described previously^39^.

### Antibodies

The following primary antibodies were used: mouse anti-TSG101 (C2, Santa Cruz Biotechnology), mouse anti-VPS4A and anti-VPS4B (A-11, Santa Cruz Biotechnology, which recognizes both isoforms), rabbit anti-HRS (C-7, Santa Cruz Biotechnology), rabbit anti-GFP (FL, Santa Cruz Biotechnology), mouse anti-HPV-16 L1 (CAMVIR-1, Santa Cruz Biotechnology. Mouse anti-16 L2 (16.D4 64-81) were kindly provided by Martin Müller while the mouse anti 16L1 33L1-7 antibody was kindly provided by Martin Sapp. Secondary antibodies conjugated to horseradish peroxidase (HRP, DAKO) or rhodamine (Molecular Probes) were used as indicated in the text.

### PsVs production

PsVs containing a luciferase reporter plasmid were generated in 293TT cells as described previously^35^. Purity and capsid protein content were determined by SDS PAGE and Coomassie Brilliant blue staining while amounts of encapsidated DNA was analysed using Real Time PCR and the copy number was quantified using a standard curve of reporter plasmid DNA.

### Infectivity Assays

HaCaT cells were seeded in 12-well plates at a density of 0.3 × 10^5^ cells/well. 72 h after siRNA transfection, cells were exposed to 100 vge/cell of luciferase reporter positive PsVs. Infection was monitored 48 h later by luminometric analysis of firefly luciferase activity using the Luciferase Assay System (Promega). For each PsV, infection with scrambled siRNA was normalized to 100%, which was then used to calculate the percentage reduction following transfection of the cells with the appropriate siRNA.

Ecdysone responsive HEK293 cells expressing inducible VPS4 were seeded in 12-well plates at a density of 0.75 × 10^5^ cells/well. 1μM ponA was then added and after 24 h the cells were infected with 100 vge/cell of luciferase reporter positive PsVs. Infection was monitored after 24 h by luminometric analysis of luciferase activity. Infection was normalized to 100% in induced cells in order to calculate the reduction in infection efficiency upon VPS4 induction. Throughout equal amounts of total cell protein extract were used in the luciferase measurements.

### GST pull-down assays

GST-tagged fusion proteins were expressed and purified as described previously^40^. HEK293 cells were transfected with VPS4A tagged GFP, VPS4B tagged DsRED and GFP tagged HRS expression plasmids and after 24 h cells were harvested and lysed in RIPA buffer. The extracts were clarified by centrifugation and then incubated with the GST fusion proteins for 2 h at room temperature. After extensive washing the bound proteins were analysed by SDS PAGE and western blotting. Direct protein-protein interactions were performed using purified His-tagged wild type VPS4B and mutant His-VPS4B 95-444 E235Q were purified on nickel agarose and then eluted in 10 mM Tris pH 8.0, 300 mM NaCl, 100 mM Sodium phosphate pH 8.0 and 200 mM imidazole. This was then added to GST alone, GST 16 L1 or GST 16 L2 bound to glutathione agarose beads and incubated for 2 h at room temperature. After washing with PBS containing 1% Triton X-100, the bound proteins were eluted by boiling in SDS PAGE sample buffer and analysed by western blotting.

### Immnunoprecipitation

HaCaT cells were seeded on 10 cm dishes at a density of 1 × 10^6^. The following day the cells were infected with 5 × 10^8^ vge PsVs per dish and kept at 4 °C for 1 h. Cells were then washed and returned to 37 °C and then harvested in RIPA at the time points indicated. Extracts were incubated with appropriate antibodies at 4 °C overnight and then incubated with protein A-conjugated sepharose beads for 1 h at room temperature. Co-immunoprecipitating L1 and L2 proteins were then detected by western blotting.

For assays in the VPS4 expressing HEK293 cells, the cells were seeded on 10 cm dishes that were pre-treated with 0.01% Poly-L-lysine (Sigma). The next day VPS4 expression was induced with 1 μM PonA for 24 h and then infected with 5 × 10^8^ vge HPV-16 PsVs per dish. Cells were harvested in RIPA buffer at different time post-infection. VPS4 was immunoprecipitated using rabbit anti-GFP antibody at 4 °C overnight and then incubated with protein A-conjugated sepharose beads for 1 h at room temperature. Co-immunoprecipitating L1 and L2 proteins were then detected by western blotting.

### Protein stability

The inducible expressing VPS4 expressing HEK293 cells were grown overnight on 0.01% Poly-L-lysine conjugated 6 well dishes. Cells were infected with PsVs and after 1 h at 4 °C the cells were immediately lysed in SDS sample buffer to monitor total input virus. Another set were then treated with trypsin for 15 mins and then immediately lysed in SDS sample buffer to detect amount of virus internalized during the first hour of incubation. The remaining cells were then incubated at 37 °C for 24 h and then treated with trypsin for 15 mins to remove any non-internalised virus. The cells were then extracted in SDS sample buffer and the levels of L1 and L2 detected by western blotting.

### Immunofluorescence

The inducible expressing VPS4 HEK293 cells were grown on 0.01% Poly-L-lysine coverslips. VPS4 induction was done by treatment with 1 uM PonA for 24 h and then infected with HPV-16 PsVs at 500 vge/cell. 7 h post-infection the cells were fixed in 3.7% paraformaldehyde for 15 min and then stained for total L1 using mouse anti L1 antibody (CAMVIR-1) or the uncoating specific anti L1 antibody 33L1-7, followed by rhodamine-conjugated rabbit anti-mouse secondary antibody. Slides were visualized using a Zeiss Axiovert 100 M microscope attached to a LSM 510 confocal unit. For quantification, the relative amount of total L1 positive cells and 33L1-7 positive cells were determined based on the total number of cells in each assay where 100 VPS4 positive cells were analysed in each experiment.

### Statistics

All experiments were performed at least three times. Statistical significance was calculated using the GraphPad prism 6 software. Two-way Anova test for the ESCRT siRNA experiments and the unpaired t-test for the assays in the VPS4 stable cell lines were used.

## Results

### A subset of ESCRT components is required for HPV infectious entry

**Figure 1:**
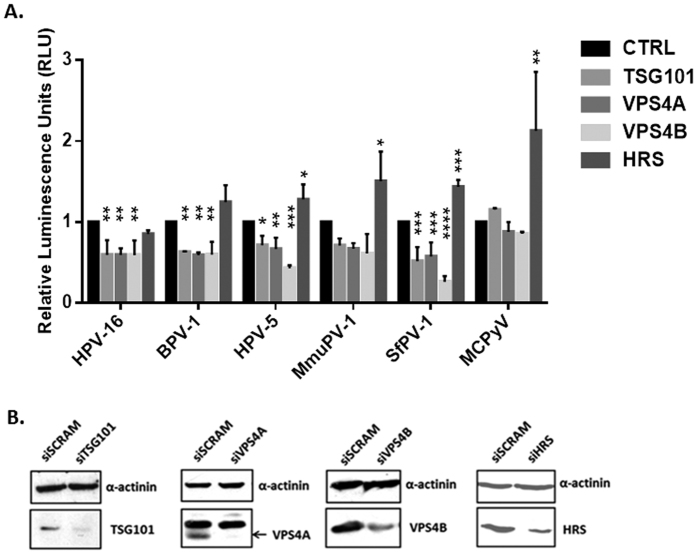
Loss of ESCRT components TSG101 and VPS4 inhibits papillomavirus infection. Panel A. HaCaT cells were transfected with siRNAs against TSG101, VPS4A, VPS4B, HRS or scrambled siRNA (siCTRL) as a control. After 72 h the cells were infected with different PsVs: HPV-16, HPV-5, BPV-1, SfPV-1, MmuPV-1, all carrying a luciferase reporter plasmid. Merkel cell polyomavirus (MCPyV) was used for comparison. After 48 h the cells were harvested and the level of luciferase activity was measured in triplicate by luminometry. The values were corrected for background luminescence and normalized to siCTRL transfected cells infected with the same type of pseudovirus. Results are expressed as means with the standard deviations of at least three independent experiments. *P ≤ 0.05; **P ≤ 0.01; ***P ≤ 0.001. Panel B. Western blot showing the levels of TSG101, VPS4A, VPS4B and HRS in HaCaT cells following transfection with the different siRNAs. Alpha-actinin was used as a loading control.

Previous studies had shown that the ESCRT component TSG101 and ESCRT-associated ALIX were required for infectious entry of HPV-16^30^,^31^. We were interested first in investigating whether the ESCRT machinery was required for infection with other papillomavirus (PV) types and, further, how the ESCRT machinery might play a role in this process. To do this we made use of the Pseudovirion (PsVs) system, where L1 and L2 are assembled into PsVs in HEK293TT cells and package a luciferase reporter plasmid^35^. In this study we used HPV-16, HPV-5, BPV-1, MmuPV-1 and SfPV-1, and as a control we also included Merkel Cell Polyomavirus (MCPyV) in the assays, which in our previous assays was found to be unaffected by TSG101 depletion^30^. HaCaT cells were first transfected with siRNAs against TSG101, VPS4A, VPS4B and HRS and after 72 h the cells were infected with the different PsVs containing luciferase. After a further 48 hrs the cells were harvested and luciferase activity monitored. The results are shown in Fig. 1 and again show an important role for TSG101 in infection with HPV-16 and BPV-1 PsVs, which is in agreement with previous reports^30^. Interestingly, we also find a requirement for TSG101 in infection with multiple other PV types, including cutaneous HPV-5 and also the animal-derived MmuPV-1 and SfPV-1. Analysis of the other ESCRT components shows a striking requirement for both VPS4A and VPS4B, which again is conserved across all the PVs analyses. Again, loss of either of these proteins has no effect on infection with MCPyV. Finally, HRS depletion appears to have no effect upon infection with the different PV PsVs, but intriguingly does appear to promote uptake of MCPyV. Taken together these studies define a subset of ESCRT-related components as being required for infection with multiple PV types.

### Both VPS4A and VPS4B interact with HPV-16 L1 and L2

**Figure 2:**
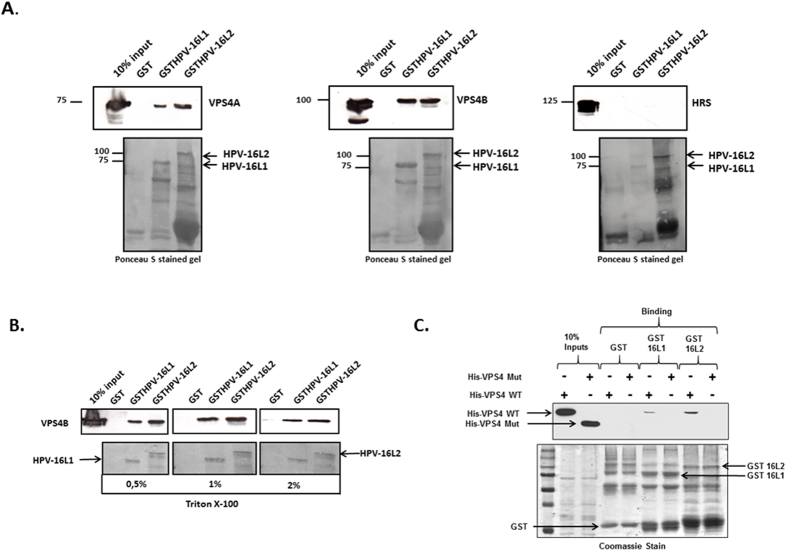
The ESCRT component VPS4 interacts with HPV-16 L1 and L2. Panel A. HEK293 cells were transfected with VPS4AGFP, VPS4BDsRED or HRSGFP expressing plasmids. 24 h after transfection the cells were lysed and extracts incubated with purified GSTHPV-16L1, GSTHPV-16L2 or GST alone as a control, for 2 h at room temperature. After extensive washing the bound protein were analyzed by Western blotting against VPS4A, VPS4B and HRS (upper panel). The lower panel shows the Ponceau stain of the nitrocellulose membrane. Panel B. HEK293 cells were transfected with VPS4BDsRED, and after 24 h the cells were lysed and extracts incubated with GSTHPV-16L1, GSTHPV-16L2 or GST alone as a control. The beads were then washed with different concentrations of Triton-X-100 (0, 5%, 1%, 2%) in PBS and bound proteins were analyzed by western blotting using anti VPS4B antibody. Panel C. The HPV-16 L1 and L2 GST fusion proteins were purified and used in interaction assays with purified His-tagged VPS4B wild type (WT) and VPS4B delta MIT mutant (Mut) proteins. After extensive washing bound VPS4 was detected by western blotting and the results shown in the upper panel. The left two lanes show the VPS4 protein inputs. The lower panel shows the Coomassie stain of the fusion proteins used in the binding assays.

Considering the importance of VPS4A and VPS4B for efficient infectious entry with multiple PV types, we were next interested in ascertaining whether there was any potential for interaction between these two proteins and the HPV capsid proteins. To do this we first performed a simple GST pull-down assay where HPV-16 L1 and L2 were expressed as GST fusion proteins and used in pull-down assays on cell extracts derived from cells over-expressing VPS4A, VPS4B and HRS. The results obtained in Fig. 2A demonstrate a clear ability of both L1 and L2 to interact with VPS4A and VPS4B, but no interaction was seen with HRS. In order to gain insight into the relative robustness of this interaction the assay was repeated with VPS4B, but the pull-downs were washed in progressively increasing concentrations of detergent. As can be seen from Fig. 2B the interactions between HPV-16 L1 and HPV-16 L2 with VPS4B are resistant to 2% Triton X-100, indicating a robust association.

To determine whether the interaction between the virion components and VPS4B was direct and involved its N-terminal MIT (microtubule interacting and trafficking) protein binding domain^38^, we performed a series of direct interaction assays using purified wild type VPS4B and a mutant VPS4B where the MIT domain was deleted, together with purified HPV-16 GST.L1 and GST.L2 fusion proteins. The results obtained are shown in Fig. 2C and demonstrate a clear direct interaction between HPV-16 L2 and VPS4B, whilst association between VPS4B and HPV-16 L1 was much weaker, albeit still detectable. Interestingly, both HPV-16 L1 and L2 failed to interact with the mutant VPS4B where the MIT domain was deleted.

**Figure 3:**
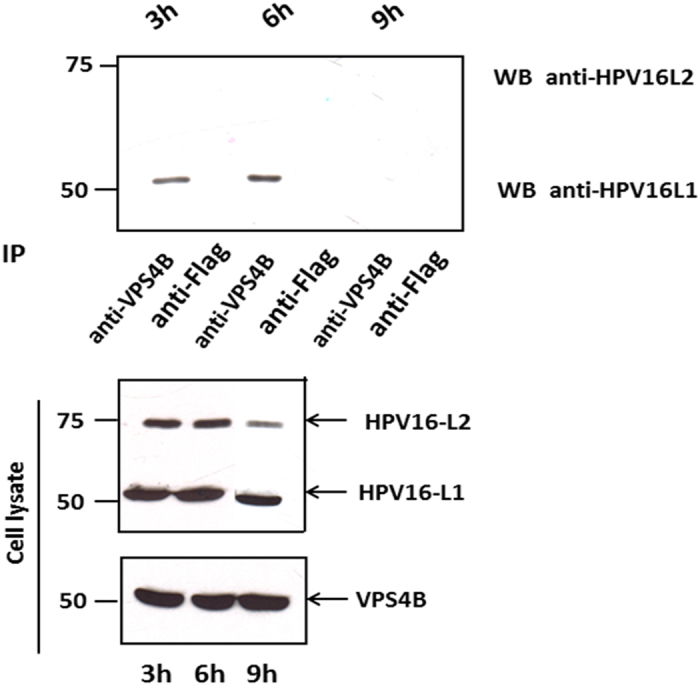
VPS4B interacts with HPV-16 L1 during viral infection. HaCaT cells were infected with HPV-16 PsVs for 1 h at 4 °C. Cells were then washed with PBS and incubated at 37 °C. At 3 h, 6 h, 9 h post-infection the cells were harvested and cell extracts incubated with anti-VPS4B or rabbit anti-Flag antibody (control) overnight at 4 °C. The immunoprecipitates were then incubated with protein A conjugated sepharose beads for 1 h at room temperature. Any co-immunoprecipitating L1 and L2 proteins were then detected by western blotting using anti-L1 and anti-L2 antibodies (top panel). The lower panel shows the protein inputs for VPS4B, L1 and L2.

We were next interested in determining whether we could detect interaction between endogenous VPS4B and the components of the viral capsid during a viral infection. To do this we infected HaCaT cells with HPV-16 PsVs and harvested the cells at 3 h, 6 h and 9 h post infection in order to determine when the interactions might take place. The extracts were then immunoprecipitated using anti-VPS4B antibody or with anti-Flag antibody as a control. The immunoprecipitates were then analysed for the presence of L1 and L2 by western blotting. As can be seen from Fig. 3, there is a clear co-immunoprecipitation of VPS4B and HPV-16 L1 at the 3 h and 6 h time points post-infection. However by 9 h post-infection the interaction appears to have been lost, suggesting that the interaction between L1 and VPS4B occurs relatively early during the infectious entry of HPV-16 PsVs. It is also noteworthy that under these particular experimental conditions, we were unable to detect interaction between L2 and VPS4B.

### HPV-16 infectious entry requires catalytically active VPS4

**Figure 4:**
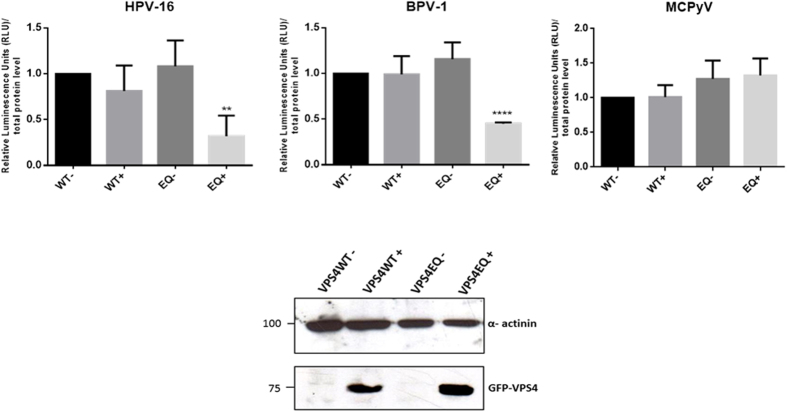
Expression of catalytically inactive VPS4EQ-GFP cells blocks infection with HPV-16 and BPV-1. Ecdysone responsive HEK 293 cells were incubated either without or with 1 μM ponA to induce expression of the VPS4WT-GFP and VPS4EQ-GFP proteins. After 24 h, the cells were infected with HPV-16, BPV-1 or MCPyV PsVs carrying a luciferase reporter plasmid. After 24 h the cells were harvested and luciferase activity assayed in triplicate by luminometry. The graphs are from at least three independent experiments and the means with standard deviations are shown. Panel B. Western blot analysis showing the expression of the VPS4WT-GFP and VPS4EQ-GFP proteins 24 h −/+ induction with 1 μM ponA. Alpha-actinin was used as loading control.

VPS4 proteins play critical roles in recycling components of the ESCRT machinery from the membranes of the MVBs for further rounds of vesicle formation^33^,^34^. This requires the VPS4 AAA-ATPase activity, defects in which give rise to large aberrant membrane structures^41^. We were therefore interested in determining whether the catalytic activity of VPS4 was likewise also required for HPV-16 infection. To investigate this we made use of previously described HEK293 cells containing an ecdysone receptor-inducible wild type VPS4 and a catalytically inactive VPS4^36^. This mutant has a single E/Q substitution in the ATPase active site (VPS4EQ) and generates a dominant negative form of VPS4. The wild type and mutant VPS4EQ were induced with ponA for 24 h and the cells were then infected with HPV-16, BPV-1 and MCPyV PsVs containing a luciferase reporter plasmid. After a further 24 h the cells were harvested and luciferase activity monitored. The results in Fig. 4 show that induction of the wild type VPS4 has minimal effect upon virus infection, however induction of the catalytically inactive dominant negative mutant form of VPS4 exerts a dramatic inhibitory effect upon infection with HPV-16 and BPV-1 PsVs. In agreement with the siRNA experiments, the dominant negative VPS4EQ mutant has no deleterious effect upon infection with MCPyV. These results demonstrate that infection with HPV-16 and BPV-1 PsVs requires the catalytic activity of VPS4, and indicates that perturbing MVB formation inhibits HPV infectious entry.

### Inactivation of VPS4 blocks capsid disassembly

**Figure 5:**
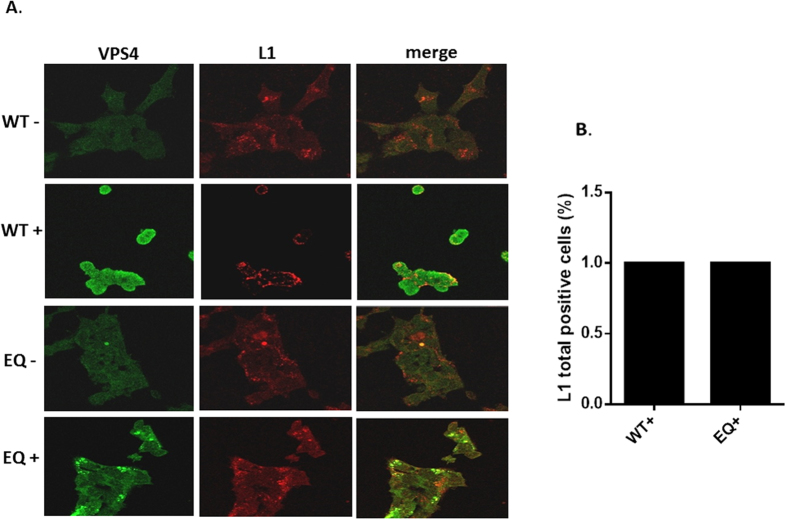
HPV-16 L1 colocalizes with the VPS4WT-GFP and VPS4EQ-GFP proteins. 293-VPS4WT-GFP and 293-VPS4EQ-GFP cells were either untreated (−) or induced (+) with 1 μM ponA and after 24 h were then infected with HPV-16 PsVs. 7 h postinfection the cells were fixed and stained for total HPV-16 L1 protein using mouse anti-L1 antibody (red). The induced VPS4WT-GFP and VPS4EQ-GFP proteins can be seen in green. Representative pictures are shown in panel A. Quantification of total L1 staining is shown in panel B.

Having shown that VPS4 ATPase activity plays an important role in the HPV-16 infectious entry pathway, we were next interested in ascertaining where the defects in HPV-16 entry might be in the cells expressing the mutant VPS4. To do this we first monitored the pattern of PsVs uptake by performing immunofluoresence for total HPV-16 L1 7 h post-infection in HEK293 cells in which the VPS4 wild type or VPS4EQ mutant were induced. The results obtained are shown in Fig. 5 and demonstrate an apparently similar distribution of HPV-16 L1 positive PsVs in the presence and absence of the wild type and mutant VPS4. Noteworthy however, is the accumulation of mutant VPS4 in larger vesicular-like structures, in agreement with previous studies^36^, where there is also a degree of L1 accumulation.

**Figure 6:**
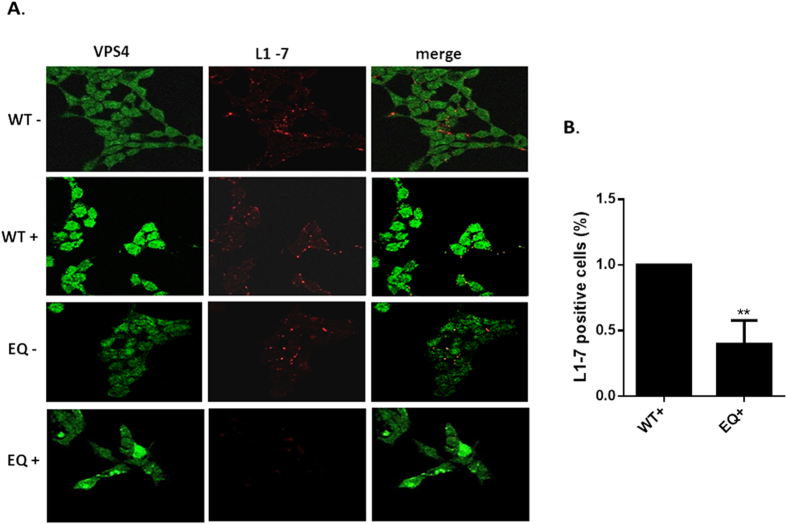
HPV-16 capsid disassembly is inhibited in cells expressing catalytically inactive VPS4EQ. 293-VPS4WT-GFP and 293-VPS4EQ-GFP cells were either untreated (−) or induced (+) with 1 μM ponA and after 24 h were infected with HPV-16 PsVs. 7 h postinfection the cells were fixed and stained using the 33L1-7 monoclonal antibody (red). The induced VPS4WT-GFP and VPS4EQ-GFP proteins can be seen in green. Representative pictures are shown in panel A. Quantification of virus disassembly based on the 33L1-7 staining is shown in panel B.

We then proceeded to investigate whether the endocytosed PsVs exhibited any defects in capsid disassembly in cells expressing the dominant negative mutant of VPS4. The VPS4 inducible HEK293 cells were again infected with HPV-16 PsVs and 7 h post infection the cells were fixed and immunofluorescence analysis performed using the 33L1-7 antibody which recognizes an epitope on L1 that is a marker of capsid disassembly^13^,^42^. The results in Fig. 6 show that capsid disassembly, indicated by 33L1-7 reactivity, occurs to a similar degree in cells that are uninduced and in cells that are expressing the wild type VPS4. In contrast, in cells expressing the VPS4EQ catalytically inactive mutant, there is a dramatic decrease in the number of cells positive for the 33L1-7 epitope. These results indicate that in the absence of functional VPS4 there is a marked defect in HPV-16 capsid disassembly.

**Figure 7:**
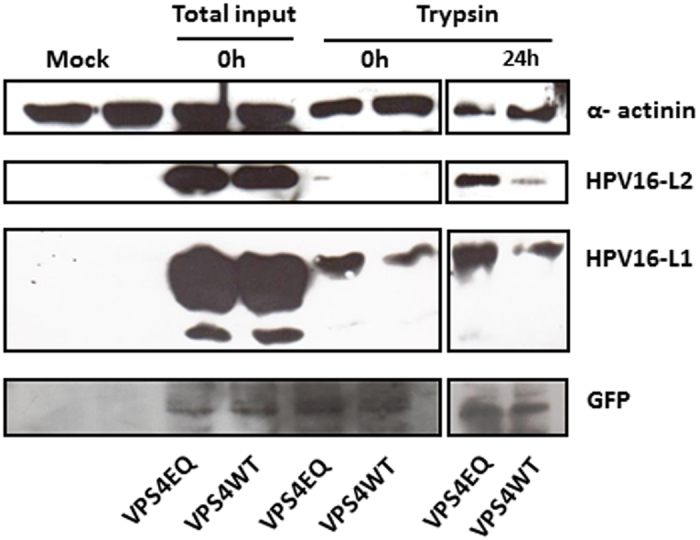
HPV-16 L1 and L2 appear more stable in mutant VPS4EQ expressing cells. The 293-VPS4WT-GFP and 293-VPS4EQ-GFP cells were induced with 1 μM ponA and after 24 h were infected with HPV-16 PsVs for 1 h at 4 °C. Cells were then washed and incubated at 37 °C for 24 h. The cells were harvested at the different times and L1 and L2 levels analysed by western blotting. The extracts are as follows: the zero time-points corresponds to the total cell lysate collected after 1 h infection (total input virus) or the lysate from cells which were first treated for 15 minutes with trypsin after 1 h infection (to detect internalized virus); the cells harvested at 24 h were also treated with trypsin 15 mins prior to harvest to remove any residual non-internalized virus and therefore represent the residual internalized L1 and L2. Alpha-actinin was used as a loading control and the induced VPS4 proteins were detected using western blotting against GFP. Note the apparent increase in L1 and L2 levels in the mutant VPS4EQ expressing cells.

Although the immunofluorescence analyses indicate no major defects in virus entry in the VPS4 mutant-expressing cells, we wanted to further address this by monitoring the levels of L1 and L2 expression 24 h post-infection. To do this the VPS4 inducible cells were infected with HPV-16 PsVs and after 1 h the cells were treated with trypsin to remove any non-internalised virus and harvested. The remaining cells were then left for 24 h and after a further round of trypsin treatment the cells were harvested and the levels of L1 and L2 protein remaining within the cells, were monitored by western blotting. The results in Fig. 7 show that L1 and L2 are still very readily detectable in the VPS4EQ mutant expressing cells at this late time-point post-infection. In contrast, the levels of L1 and L2 are reduced in the wild type VPS4 expressing cells. These results appear to indicate no major defect in the entry of HPV-16 PsVs into the VPS4EQ mutant expressing cells. Rather, they suggest that entry is either more efficient than in the wild type VPS4-expressing cells, or more likely, that the processing of L1 and L2 in the mutant VPS4EQ-expressing cells is in some way perturbed, resulting in the maintenance of higher levels of L1 and L2.

### HPV-16 L1 and L2 form a complex with VPS4 *in vivo*

**Figure 8:**
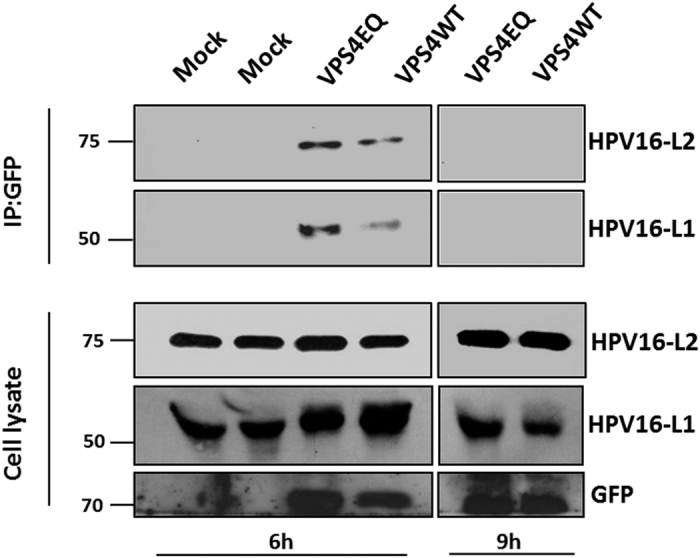
Both HPV16 L1 and HPV16 L2 interact with VPS4WT-GFP and VPS4EQ-GFP during virus infection. The 293-VPS4WT-GFP and 293-VPS4EQ-GFP cells were either untreated (−) or induced (+) with 1 μM ponA and after 24 h were infected with HPV-16 PsVs. At 6 h and 9 h post infection the cells were harvested and cell extracts incubated with rabbit anti-GFP antibody overnight at 4 °C. The next day immunoprecipitates were collected on protein A conjugated sepharose beads for 1 h at room temperature. The co-immunoprecipitating L1 and L2 proteins were then detected by western blotting using mouse anti-L1 and mouse anti-L2 antibodies (top panel). The lower panel shows the inputs for HPV16 L1, HPV16 L2 and VPS4 (GFP).

Having found evidence in Fig. 3 that HPV-16 L1 could interact with VPS4 during the course of infectious entry, we also wanted to determine whether this also occurred with the VPS4EQ mutant. Again we used the VPS4 inducible HEK293 cells and infected them with HPV-16 PsVs. The cells were harvested at 6 h and 9 h post-infection and immunoprecipitated with rabbit anti-GFP antibody to immunoprecipitate the GFP-tagged VPS4 protein. Co-immunoprecipitating L1 and L2 was then detected by western blotting and the results obtained are shown in Fig. 8. In agreement with the results in Fig. 3, L1 co-immunoprecipitates with VPS4 at the 6 h time point post-infection, but this is not visible 9 h post-infection, again suggesting an interaction occurring early in infection. Interestingly, there appears to be a somewhat stronger association between L1 and the mutant VPS4EQ than with the wild type protein, although this needs to be tempered by the fact that there is somewhat more mutant VPS4 present than the wild type in this experiment. In addition, this analysis shows clear evidence of association between HPV-16 L2 and the wild type and mutant forms of the VPS4 protein. Taken together these results demonstrate that incoming HPV-16 PsVs complex with VPS4, and that this complex requires catalytically active VPS4 protein to ensure the completion of successful virus entry and capsid disassembly.

## Discussion

In this study we have investigated how the ESCRT machinery contributes towards HPV infection. We provide evidence of an important role for this complex in infection with multiple PV types, suggesting that this plays an evolutionarily conserved role in PV infectious entry. Furthermore, we provide evidence for a critical role for the VPS4 component of the ESCRT machinery, since loss of its expression or inactivation of its ATPase activity through the generation of a dominant negative mutation results in a dramatic reduction in infection with HPV-16 PsVs. The defect appears to be at an early step in infection, and corresponds to a stage prior to capsid disassembly. At this stage we cannot say whether the effects of knock down of VPS4 on virus infection are indirect due to a general perturbation of the endocytic machinery, or whether there is a more specific role in HPV entry. Intriguingly however, both L1 and L2 appear to be capable of independent direct association with VPS4, and can be found in complex with VPS4 at early times post-infection. These results suggest that VPS4 performs a function that favours capsid disassembly and allows a continuation of the later stages of infectious entry.

Recent studies have provided some compelling insights into a potential role for the ESCRT machinery and MVB formation in HPV infectious entry. This was initially triggered by studies showing the presence of incoming virions in endosomes containing MVBs^12^, and a requirement for TSG101 for efficient infectious entry^30^. More recently, the ESCRT accessory protein ALIX was also shown to play an import role in viral trafficking and capsid disassembly^31^. In the present study we found an important role for ESCRT accessory protein VPS4 for infection with HPV-16 PsVs. In addition, whilst previous studies had shown that TSG101 was also required for infection with BPV-1 PsVs^30^ here we show an important role for both TSG101 and VPS4 for infection with cutaneous HPV-5 PsVs and also mouse MmuPV-1 and rabbit SfPV-1 indicating that the ESCRT machinery most likely plays a role in infection with multiple PV types, and demonstrates an evolutionarily conserved step in the infectious entry of multiple PV types.

Identifying the two VPS4 isoforms, VPS4A and VPS4B AAA-ATPase proteins, as important elements in HPV infection is particularly exciting. These proteins play critical roles in separating components of the ESCRT machinery from the MVBs that form within late endosomes. Both proteins form heteromeric complexes and defects in either results in aberrant membrane E compartments^43^. Whilst we have not attempted to differentiate between these two isoforms as to whether they exert different functions during virus infection however, based on the similarity of effects in the siRNA knock down assays, it seems likely that their respective functions will be similar. An essential requirement for VPS4 activity is a functional ATPase. By using a cell line which expresses a dominant negative mutation within the ATPase active site, we find that the VPS4 ATPase activity is also essential for HPV-16 PsVs infection. Interestingly, cells expressing this mutant display major defects in MVB maturation^38^, and currently it is unclear whether the defect in infection is simply a side-effect of an alteration in MVB formation and endosomal maturation. However, it is tempting to suggest that the effects of VPS4 are rather more direct, and there is some compelling evidence to support this. First, we find clear evidence for direct interaction of the L1 and L2 capsid proteins with VPS4 *in vitro*, suggesting that both L1 and L2, might be bound by VPS4. This interaction requires an intact MIT domain, but currently we have found no evidence for the presence of a classical MIT interaction motif^44^,^45^ in either L1 or L2. Therefore further studies will be required to characterize the mechanism of interaction and to also determine whether L1 or L2 can affect the ability of VPS4 to interact with its cellular binding partners.

There is also clear evidence of interaction of L1 and L2 with VPS4 during HPV-16 PsVs infectious entry. Most interestingly, this interaction appears to occur at relatively early time points post-infection, with interaction between L1 and VPS4 detectable at 3 h and 6 h post-infection but by 9 h post infection the interaction was not detectable. The lack of interaction at later time points would suggest that incoming virions pass through this point in the entry pathway relatively early during infection, and this is in agreement with previous studies which have shown that virion trafficking can be rapid^46^.

Although we could only see evidence of L1 interacting with endogenous VPS4 in HaCaT cells, both L1 and L2 were found in complex with VPS4 in the HEK293 cells expressing GFP-tagged forms of the wild type and catalytically inactive forms of VPS4. The reason for this difference is unclear, but might be a reflection either of the very high levels of infection obtained in HEK293 cells or the higher levels of VPS4 expression in the inducible cell lines. Further studies will be needed to ascertain whether L1 and L2 are associated with VPS4 as part of a complex, and what specific role this may play in the infection process.

Having found an essential role for VPS4 in efficient infectious entry, we were next interested in determining where the interaction was required. Clearly interaction occurs quite early. Analysis of virus uptake by immunofluorescence or trypsin digestion of cell surface-bound virions seems to indicate that uptake of the virus is not affected by loss of VPS4 ATPase activity. However there does seem to be accumulation of incoming PsVs in the aberrant membrane structures that form within cells with defective VPS4, and this appears to be accompanied by an increase in the stability of the incoming L1 and L2 proteins, suggesting a block in the endocytic processing of these two viral proteins. Most intriguingly we see that the defect in infection caused by expression of the VPS4EQ mutant appears to occur at an early stage prior to capsid disassembly. Using the 33L1-7 antibody as a marker of capsid disassembly, we see clear evidence of a dramatic decrease in the ability to detect capsid disassembly in cells that are expressing the catalytically inactive mutant form of VPS4. This suggests that the VPS4 ATPase activity is required for capsid disassembly and subsequent trafficking of L1 to the lysosomal compartments. It is interesting to note that previous studies had shown a requirement for cyclophilins in the separation of L1 from L2^13^, a step in infectious entry that was also blocked by using broad-spectrum ATPase inhibitors^47^. Therefore it is possible that VPS4 is also playing a role in ensuring the proper separation of L1 from L2 during virus infection.

Many other viruses have been shown to utilize components of the ESCRT machinery for various aspects of their life cycles^48^, but in the majority of cases this involves events related to membrane fusion or fission with enveloped viruses. Obviously the role of the ESCRT machinery in non-enveloped virus infection is very different. The studies presented here and elsewhere^31^ suggest that MVB formation is an important step in HPV infectious entry. Furthermore, we provide evidence that this plays an important role during the early stages of infection, prior to capsid disassembly. Finally, we propose that the ATPase activity of VPS4 might be playing a role in ensuring efficient partitioning of L1 from L2, and thereby promoting successful capsid disassembly. Taken together this suggests that there may be two distinct roles for the ESCRT machinery, one involving endosome maturation and the other potentially in aiding capsid uncoating.

## Additional Information

**How to cite this article:** Broniarczyk, J. *et al*. The VPS4 component of the ESCRT machinery plays an essential role in HPV infectious entry and capsid disassembly. *Sci. Rep.* **7**, 45159; doi: 10.1038/srep45159 (2017).

**Publisher's note:** Springer Nature remains neutral with regard to jurisdictional claims in published maps and institutional affiliations.

## References

[b1] zur Hausen H. Papillomavirus infections—a major cause of human cancers. Biochim. Biophys. Acta 1288(2), F55–F78 (1996). 8876633 10.1016/0304-419x(96)00020-0

[b2] zur Hausen H. Papillomaviruses and cancer: from basic studies to clinical application. Nat. Rev. Cancer 2(5), 342–50 (2002). 12044010 10.1038/nrc798

[b3] Bouvard V. . WHO International Agency for Research on CancerMonograph Working Group. A review of human carcinogens—Part B: biological agents. Lancet Oncol. 10(4), 321–2 (2009). 19350698 10.1016/s1470-2045(09)70096-8

[b4] Doorbar J. . The biology and life-cycle of human papillomaviruses. Vaccine Suppl 5, F55–F70 (2012). 10.1016/j.vaccine.2012.06.083 23199966

[b5] Ganti K. . The Human Papillomavirus E6 PDZ Binding Motif: From Life Cycle to Malignancy. Viruses 7(7), 3530–51 (2015). 26147797 10.3390/v7072785 PMC4517114

[b6] Thomas M. . Human papillomaviruses, cervical cancer and cell polarity. Oncogene 27(55), 7018–30 (2008). 19029942 10.1038/onc.2008.351

[b7] Buck C. B. . Arrangement of L2 within the papillomavirus capsid. J. Virol. 82, 5190–5197 (2008). 18367526 10.1128/JVI.02726-07 PMC2395198

[b8] Day P. M., Lowy D. R. & Schiller J. T. Heparan sulfate-independent cell binding and infection with furin-precleaved papillomavirus capsids. J. Virol. 82, 12565–12568 (2008). 18829767 10.1128/JVI.01631-08 PMC2593329

[b9] Joyce J. G. . The L1 major capsid protein of human papillomavirus type 11 recombinant virus-like particles interacts with heparin and cell-surface glycosaminoglycans on human keratinocytes. J. Biol. Chem. 274, 5810–5822 (1999). 10026203 10.1074/jbc.274.9.5810

[b10] Giroglou T., Florin L., Schafer F., Streeck R. E. & Sapp M. Human papillomavirus infection requires cell surface heparan sulfate. J. Virol. 75, 1565–1570 (2001). 11152531 10.1128/JVI.75.3.1565-1570.2001 PMC114064

[b11] Yang R., Day P. M., Yutzy W. H., Lin K. Y., Hung C. F. & Roden R. B. Cell surface-binding motifs of L2 that facilitate papillomavirus infection. J. Virol. 77, 3531–35412003 (2003). 12610128 10.1128/JVI.77.6.3531-3541.2003 PMC149523

[b12] Schelhaas M. . Entry of human papillomavirus type 16 by actin-dependent, clathrin- and lipid raft-independent endocytosis. PLoS Pathog. 8, e1002657 (2012). 22536154 10.1371/journal.ppat.1002657 PMC3334892

[b13] Bienkowska-Haba M., Williams C., Kim S. M., Garcea R. L. & Sapp M. Cyclophilins facilitate dissociation of the human papillomavirus type 16 capsid protein L1 from the L2/DNA complex following virus entry. J. Virol. 86, 9875–87 (2012). 22761365 10.1128/JVI.00980-12 PMC3446629

[b14] Schiller J. T., Day P. M. & Kines R. C. Current understanding of the mechanism of HPV infection. Gynecol. Oncol. 118 (1 Suppl), S12–7 (2010). 20494219 10.1016/j.ygyno.2010.04.004 PMC3493113

[b15] Buck C. B., Day P. M. & Trus B. L. The papillomavirus major capsid protein L1. Virology 445(1–2), 169–74 (2013). 23800545 10.1016/j.virol.2013.05.038 PMC3783536

[b16] Bronnimann M. P., Chapman J. A., Park C. K. & Campos S. K. A transmembrane domain and GxxxG motifs within L2 are essential for papillomavirus infection. J. Virol. 87(1), 464–73 (2013). 23097431 10.1128/JVI.01539-12 PMC3536380

[b17] DiGiuseppe S. . Topography of the Human Papillomavirus Minor Capsid Protein L2 during Vesicular Trafficking of Infectious Entry. J. Virol. 89(20), 10442–52 (2015). 26246568 10.1128/JVI.01588-15 PMC4580179

[b18] Lipovsky A. . Genome-wide siRNA screen identifies the retromer as a cellular entry factor for human papillomavirus. Proc. Natl. Acad. Sci. USA 110, 7452–7457 (2013). 23569269 10.1073/pnas.1302164110 PMC3645514

[b19] Popa A. . Direct Binding of Retromer to Human Papillomavirus Type 16 Minor Capsid Protein L2 Mediates Endosome Exit during Viral Infection. PLoS Pathog 11, e1004699 (2015). 25693203 10.1371/journal.ppat.1004699 PMC4334968

[b20] Bergant Marušič M., Ozbun M. A., Campos S. K., Myers M. P. & Banks L. Human papillomavirus L2 facilitates viral escape from late endosomes via sorting nexin 17. Traffic 13(3), 455–67 (2012). 22151726 10.1111/j.1600-0854.2011.01320.x PMC3276720

[b21] Pim D., Broniarczyk J., Bergant M., Playford M. P. & Banks L. A Novel PDZ Domain Interaction Mediates the Binding between Human Papillomavirus 16 L2 and Sorting Nexin 27 and Modulates Virion Trafficking. J. Virol. 89, 10145–55. (2015). 26202251 10.1128/JVI.01499-15 PMC4580170

[b22] Day P. M., Thompson C. D., Schowalter R. M., Lowy D. R. & Schiller J. T. Identification of a role for the trans-Golgi network in human papillomavirus 16 pseudovirus infection. J. Virol. 87, 3862–70 (2013). 23345514 10.1128/JVI.03222-12 PMC3624235

[b23] Day P. M., Baker C. C., Lowy D. R. & Schiller J. T. Establishment of papillomavirus infection is enhanced by promyelocytic leukemia protein (PML) expression. Proc Natl Acad Sci USA 101, 14252–14257 (2004). 15383670 10.1073/pnas.0404229101 PMC521143

[b24] Pyeon D., Pearce S. M., Lank S. M., Ahlquist P. & Lambert P. F. Establishment of human papillomavirus infection requires cell cycle progression. PLoS Pathog 5, e1000318 (2009). 19247434 10.1371/journal.ppat.1000318 PMC2642596

[b25] Aydin I. . Large scale RNAi reveals the requirement of nuclear envelope breakdown for nuclear import of human papillomaviruses. PLoS Pathog. 10(5), e1004162 (2014). 24874089 10.1371/journal.ppat.1004162 PMC4038628

[b26] Broniarczyk J., Massimi P., Bergant M. & Banks L. Human papillomavirus infectious entry and trafficking is a rapid process. J. Virol. 89, 8727–32 (2015). 26063434 10.1128/JVI.00722-15 PMC4524062

[b27] Huotari J. & Helenius A. Endosome maturation. EMBO J. 30(17), 3481–3500 (2011). 21878991 10.1038/emboj.2011.286 PMC3181477

[b28] Katzmann D. J., Odorizzi G. & Emr S. D. Receptor downregulation and multivesicular-body sorting. Nat. Rev. Mol. Cell. Biol. 3(12), 893–905 (2002). 12461556 10.1038/nrm973

[b29] Saksena S., Sun J., Chu T. & Emr S. D. ESCRTing proteins in the endocytic pathway. Trends Biochem. Sci. 32(12), 561–573 (2007). 17988873 10.1016/j.tibs.2007.09.010

[b30] Broniarczyk J., Bergant M., Goździcka-Józefiak A. & Banks L. Human papillomavirus infection requires the TSG101 component of the ESCRT machinery. Virology 460–461, 83–90 (2014). 10.1016/j.virol.2014.05.005 25010273

[b31] Gräßel L. . The CD63-Syntenin-1 Complex Controls Post-Endocytic Trafficking of Oncogenic Human Papillomaviruses. Sci. Rep. 6, 32337, 10.1038/srep32337 (2016). 27578500 PMC5006017

[b32] Wollert T. & Hurley J. H. Molecular mechanism of multivesicular body biogenesis by ESCRT complexes. Nature 464(7290), 864–9 (2010). 20305637 10.1038/nature08849 PMC2851844

[b33] Stuchell-Brereton M. D., Skalicky J. J., Kieffer C., Karren M. A., Ghaffarian S. & Sundquist W. I. ESCRT-III recognition by VPS4 ATPases. Nature 449(7163), 740–4 (2007). 17928862 10.1038/nature06172

[b34] Adell M. A., Migliano S. M. & Teis D. ESCRT-III and Vps4: a dynamic multipurpose tool for membrane budding and scission. FEBS J. 10.1111/febs.13688 (2016). 26910595

[b35] Buck C. B., Pastrana D. V., Lowy D. R. & Schiller J. T. Generation of HPV pseudovirions using transfection and their use in neutralization assays. Methods Mol Med 119, 445–462 (2005). 16350417 10.1385/1-59259-982-6:445

[b36] Crump C. M., Yates C. & Minson T. Herpes simplex virus type 1 cytoplasmic envelopment requires functional Vps4. J. Virol. 81(14), 7380–7 (2007). 17507493 10.1128/JVI.00222-07 PMC1933334

[b37] Graham F. L. & van der Eb A. J. A new technique for the assay of infectivity of human adenovirus 5 DNA. Virology 52(2), 456–67 (1973). 4705382 10.1016/0042-6822(73)90341-3

[b38] Scott A. . Structural and mechanistic studies of VPS4 proteins. EMBO J. 24(20), 3658–69 (2005). 16193069 10.1038/sj.emboj.7600818 PMC1276703

[b39] Janvier K., Pelchen-Matthews A., Renaud J. B., Caillet M., Marsh M. & Berlioz-Torrent C. The ESCRT-0 component HRS is required for HIV-1 Vpu-mediated BST-2/tetherin down-regulation. PLoS Pathog. 7(2), e1001265 (2011). 21304933 10.1371/journal.ppat.1001265 PMC3033365

[b40] Thomas M., Massimi P. & Banks L. HPV-18 E6 inhibits p53 DNA binding activity regardless of the oligomeric state of p53 or the exact p53 recognition sequence. Oncogene 13(3), 471–80 (1996). 8760288

[b41] Fujita H. . A dominant negative form of the AAA ATPase SKD1/VPS4 impairs membrane trafficking out of endosomal/lysosomal compartments: class E vps phenotype in mammalian cells. J. Cell. Sci. 116 (Pt 2), 401–14 (2003). 12482925 10.1242/jcs.00213

[b42] Sapp M., Kraus U., Volpers C., Snijders P. J., Walboomers J. M. & Streeck R. E. Analysis of type-restricted and cross-reactive epitopes on virus-like particles of human papillomavirus type 33 and in infected tissues using monoclonal antibodies to the major capsid protein. J. Gen. Virol. 75 (Pt 12), 3375–83 (1994). 7996132 10.1099/0022-1317-75-12-3375

[b43] Scheuring S. . Mammalian cells express two VPS4 proteins both of which are involved in intracellular protein trafficking. J Mol Biol. 312(3), 469–80 (2001). 11563910 10.1006/jmbi.2001.4917

[b44] Monroe N. & Hill C. P. Meiotic Clade AAA ATPases: Protein Polymer Disassembly Machines. J Mol Biol. 428 (9 Pt B), 1897–911 (2016). 26555750 10.1016/j.jmb.2015.11.004 PMC4860049

[b45] Christ L., Raiborg C., Wenzel E. M., Campsteijn C. & Stenmark H. Cellular Functions and Molecular Mechanisms of the ESCRT Membrane-Scission Machinery. Trends Biochem Sci. 42(1), 42–56 (2017). 27669649 10.1016/j.tibs.2016.08.016

[b46] Broniarczyk J., Massimi P., Bergant M. & Banks L. Human Papillomavirus Infectious Entry and Trafficking Is a Rapid Process. J Virol. 89(17), 8727–32 (2005). 10.1128/JVI.00722-15 PMC4524062 26063434

[b47] Müller K. H. . Inhibition by cellular vacuolar ATPase impairs human papillomavirus uncoating and infection. Antimicrob. Agents Chemother. 58(5), 2905–11 (2014). 24614368 10.1128/AAC.02284-13 PMC3993236

[b48] Votteler J. & Sundquist W. I. Virus budding and the ESCRT pathway. Cell Host Microbe 14(3), 232–41 (2013). 24034610 10.1016/j.chom.2013.08.012 PMC3819203

